# The Auchenorrhyncha fauna of peat bogs in the Austrian part of the Bohemian Forest (Insecta, Hemiptera)

**DOI:** 10.3897/zookeys.319.4324

**Published:** 2013-07-30

**Authors:** Werner E. Holzinger, Lydia Schlosser

**Affiliations:** 1 Ökoteam – Institute for Animal Ecology and Landscape Planning, Bergmanngasse 22, 8010 Graz, Austria

**Keywords:** Auchenorrhyncha, Fulgoromorpha, Cicadomorpha, peat bogs, wetland, species composition, Bohemian Forest, Austria

## Abstract

The first overview on the Auchenorrhyncha fauna of peat bogs of the Austrian Bohemian Forest is presented. Seven oligotrophic peat bog sites were studied in 2011 by suction sampler (“G-Vac”) and 93 Auchenorrhyncha species (with 7465 adult specimens) were recorded. Eleven species (about 18 % of the individuals) are tyrphobiontic or tyrphophilous. The relative species abundance plot is not very steep; the six most abundant species represent 50 % of the individuals. The most common species is *Conomelus anceps* (17 % of the individuals). Compared to the whole Austrian Auchenorrhyncha fauna, the fauna of peat bogs comprises distinctly more univoltine species and more species hibernating in nymphal stage. Densities of adult Auchenorrhyncha in peat bogs are low in spring (about 10–60 individuals per m²) and high in July, with up to 180 (±50) individuals per m². Disturbed peat bogs have higher species numbers and higher Auchenorrhyncha densities in total, but lower numbers and densities in peat bog specialists.

## Abstract (de)

Diese Studie gibt erstmals einen Überblick über die Zikadenfauna der Moore des österreichischen Anteils des Böhmerwaldes. Sieben Moorflächen wurden 2011 mittels Saugfang quantitativ untersucht. Insgesamt konnten 93 Zikadenarten (in 7465 adulten Individuen) festgestellt werden. Elf Arten (ca. 18 % der Individuen) sind tyrphobiont oder tyrphophil. Die Gesamtdominanzkurve ist nicht sehr steil; die sechs häufigsten Arten repräsentieren 50 % aller Individuen, die häufigste Art ist *Conomelus anceps* (17 % der Individuen). Vergleicht man die Zikadenfauna der Moore mit der Zikadenfauna Österreichs, so weist erstere deutlich mehr univoltine Arten und mehr Larvalüberwinterer auf. Phänologisch betrachtet sind die Dichten adulter Zikaden in Mooren im Frühling niedrig (ca. 10–60 Individuen pro m²) und im Juli hoch (bis 180 ± 50 Individuen pro m²). Gestörte Moorlebensräume haben höhere Artenzahlen und höhere Zikadendichten, aber die Artenzahl und Individuendichte der Moorspezialisten ist deutlich geringer als in ungestörten Mooren.

## Introduction

Peat bogs are characterized by very wet, acidic and oligotrophic conditions, and their soil is of organic origin. They are among the most threatened habitats in Central Europe, due to dewatering, peat mineralization, land reclamation, afforestation, nutrient contamination and recently by climate change. Within the last century, over 90 % of all peat bogs in Austria were devastated or completely destroyed (Niedermair et al. 2010).

Auchenorrhyncha are among the most abundant animal groups in peatlands. The majority of species is stenoecious, specialized on both habitat conditions and host plants (Nickel et al. 2002, Nickel 2003; see Table 3). With a few exceptions (Leising 1977, Holzinger 1995, 2000, Holzinger and Novotny 1998) the Auchenorrhyncha fauna of Austrian peat bogs is poorly studied, and the hopper fauna of the granite and gneiss highlands of the Austrian Bohemian Forest is completely unknown. Here we present the first overview on the peat bog fauna of this area and give some data on the Auchenorrhyncha communities of Central European peat bogs (see also Schlosser 2012, Schlosser and Holzinger 2012).

## Methods and materials

Seven typical oligotrophic peat bog sites of the Bohemian Forest were studied in 2011. Quantitative samples were taken monthly (from May until September) by a suction sampler (“G-Vac”, see Stewart 2002). Each sample was taken by walking slowly through the sampling site and performing 100 “touchdowns” with the suction sampler nozzle (Ø 12 cm). Thus one sample represents the fauna of 1.1 m². Three samples per date and site were taken, thus the data set contains a total of (7 sites × 5 dates × 3 samples =) 105 samples.

### Study sites

**Figure 1.**
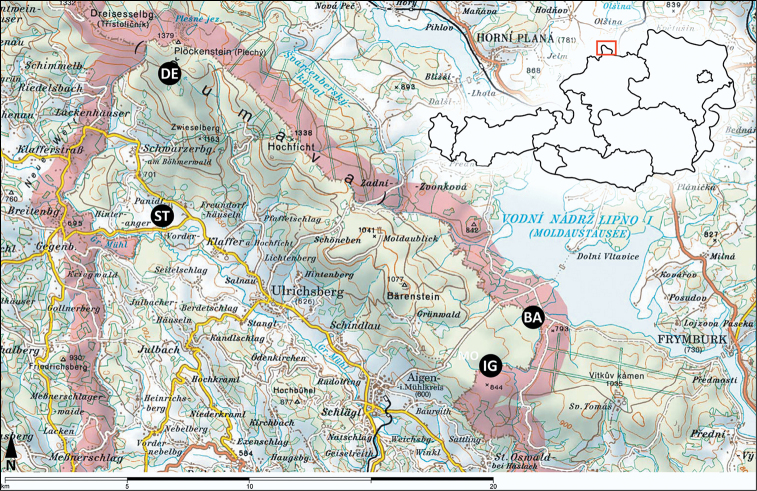
Location of the sampling sites in the Bohemian Forest (Upper Austria), overview. Samling sites: DE = Deutsches Haidl, ST = Stadlau, BA = Bayrische Au, IG = Moor am Iglbach. Map source: AMAP 3D.

The study sites are located in the very north of Upper Austria, close to the German and Czech border. They are shown in Fig. 1 and characterized in Table 1 and 2.

**Table 1.** Study sites, coordinates and sampling dates on these sites (according to the Upper Austrian environmental lawyer, unpublished).

**Code**	**Site name**	**Coordinates**	**Altitude**	**Area (ha)**	**Site description**	**Sampling dates**
BA	Bayrische Au	48°40'49"N, 14°03'32"E	720 m	33.8	oligotrophic peat bog of national importance (Steiner 1992)	13.5., 22.6., 27.7., 22.8., 24.9.2011
ST-1	Stadlau 1	48°42'29"N, 13°51'12"E	610 m	7.1	dewatered, partially eroded and heavily nutrient contaminated remnants of a formerly large peat bog area	17.5., 20.6., 25.7., 21.8., 23.9.2011
ST-2	Stadlau 2	48°42'23"N, 13°51'18"E	610 m			
ST-3	Stadlau 3	48°42'32"N, 13°51'15"E	610 m			
IG-1	Iglbach-Moor 1	48°39'10"N, 14°01'44"E	800 m	3.9	drained peat bog complex, oligotrophic to mesotrophic, partially still very wet	19.5., 22.6., 26.7., 20.8., 24.9.2011
IG-2	Iglbach-Moor 2	48°39'09"N, 14°01'30"E	800 m			
DE	Deutsches Haidl	48°45'43"N, 13°51'13"E	1242 m	2.8	acidic oligotrophic peat bog of international importance (Steiner 1992); sphagnum moss–spruce forest with a large central area covered by *Carex limosa* and *Sphagnum majus*	18.5., 25.6., 27.7., 22.8., 25.9.2011

**Table 2.** Vegetation and management of the study sites (according to the Upper Austrian environmental lawyer, unpublished).<br/>

**Code**	**Site name**	**Vegetation**	**Site management**
BA	Bayrische Au	Patchy mixture of Phalaridetum arundinaceae, Caricetum rostratae, Caricetum gracilis, Caricetum nigrae, Sphagnetum magellanici	parts of the peat bog formerly used for peat-ditching; no management today
ST-1	Stadlau 1	Molinion, Sphagnetum magellanici	some years grazed (cattle) but not in 2011
ST-2	Stadlau 2	Caricetum nigrae, Caricetum rostratae, Junco-Molinietum	some years grazed (cattle) but not in 2011
ST-3	Stadlau 3	Junco-Molinietum	mowing once a year (July), grazed (cattle)
IG-1	Iglbach-Moor 1	Caricetum rostratae	no management
IG-2	Iglbach-Moor 2	Caricetum rostratae	no management
DE	Deutsches Haidl	Caricetum limosae, Sphagnetum magellanici, Sphagno girgensohnii-Piceetum	no management

## Results and discussion

**Figure 2.**
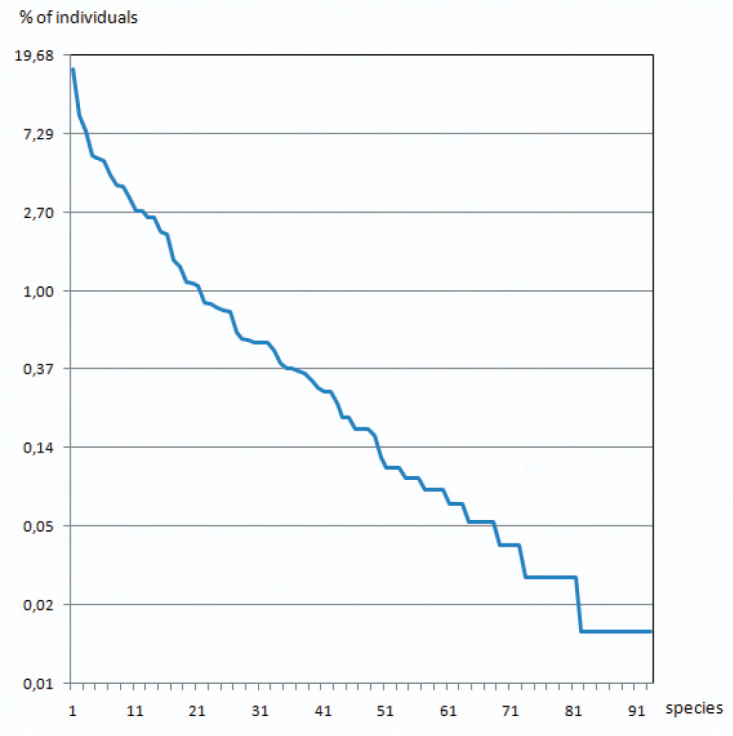
Species abundance ranking. The species are ordered by their relative abundance (descending).

A total number of 93 Auchenorrhyncha species (7465 adult specimens) were collected and identified (Tables 3 and 4﻿). The most abundant species is *Conomelus anceps* representing almost 17 per cent of the total number of specimens, followed by *Jassargus pseudocellaris* (5.5 %), *Muellerianella extrusa* (9.2 %), *Sorhoanus xanthoneurus* (7.6 %) and *Macustus grisescens* (5.2 %). The relative species abundance plot ﻿(Fig. 2) is not very steep; the six most abundant species represent only 50 % of the total individuals, and the 75 % mark is reached at species number 14.

**Table 3.** Overview on Auchenorrhyncha collected in seven peat bogs in the Austrian part of the Bohemian Forest. Abbreviations: BA = Bayrische Au, ST = Stadlau, IG = Moor am Iglbach, DE = Deutsches Haidl.

	**BA**	**ST-1**	**ST-2**	**ST-3**	**IG-1**	**IG-2**	**DE**	**Total**
Total number of adult specimens	1389	735	667	1724	891	1446	613	7465
Total number of taxa	31	29	30	50	44	47	19	93
Number of tyrphobiontic and tyrphophilous individuals	50	80	155	26	154	290	580	1333
Number of tyrphobiontic and tyrphophilous species	5	5	4	5	7	5	3	11
Percentage of peat bog specialists (individuals)	3.6	10.8	23.2	1.5	17.2	20.0	94.6	17.9
Percentage of peat bog specialists (species)	16.1	17.2	13.3	10.0	15.9	10.6	15.8	11.8

**Table 4.** Auchenorrhyncha species of peat bogs in the Austrian part of the Bohemian Forest. The species are grouped into ecological types after Holzinger (2009), except for tyrphobiontic and tyrphophilous species identified after Nickel et al. (2002). Within one type, species are sorted in descending number. Abbreviations: BA = Bayrische Au, ST = Stadlau, IG = Moor am Iglbach, DE = Deutsches Haidl; ind. = individuals; rel. abd. = relative abundance; RL A = threat status according to the Austrian Red List (Holzinger 2009): LC = least concern, DD = data deficient, NT = near threatened, VU = vulnerable, EN = endangered, CR = critically endangered.

**No.**	**Species**	**Total number of specimens/percentage of total abundance of sampling site**							**Total ind.**	**rel. abd. (%)**	**RL A**
		**BA**	**ST-1**	**ST-2**	**ST-3**	**IG-1**	**IG-2**	**DE**			
**Tyrphobiontic species**											
1	*Sorhoanus xanthoneurus* (Fieber, 1869)					1 0,1		567 92,5	568	7.6	CR
2	*Kelisia vittipennis* (J. Sahlberg, 1868)	1 0,1	18 2,4	87 13		24 2,7	150 10,4		280	3.8	VU
3	*Stroggylocephalus livens* (Zetterstedt, 1840)	6 0,4							6	0.1	CR
4	*Cixius similis* Kirschbaum, 1868							1 0,2	1	<0.1	VU
**Tyrphophilous species**											
5	*Sorhoanus assimilis* (Fallén, 1806)	16 1,2	48 6,5	55 8,2	1 0,1	33 3,7	25 1,7	12 2	190	2.5	VU
6	*Paradelphacodes paludosa* (Flor, 1861)	15 1,1	1 0,1	6 0,9	2 0,1	61 6,8	74 5,1		159	2.1	EN
7	*Oncodelphax pullula* (Boheman, 1852)	12 0,9	9 1,2	7 1		17 1,9	15 1		60	0.8	EN
8	*Cicadula saturata* (Edwards, 1915)		4 0,5			11 1,2	26 1,8		40	0.5	
9	*Macrosteles ossiannilssoni* Lindberg, 1954				15 0,9				15	0.2	NT
10	*Kelisia ribauti* Wagner, 1938 „boreomontan“				1 0,1	7 0,8			8	0.1	EN
11	*Kelisia ribauti* Wagner, 1938 „mediterran“				7 0,4				7	0.1	EN
**Hygrophilous grassland species**											
12	*Conomelus anceps* (Germar, 1821)	847 61	16 2,2	177 26,5	22 1,3	17 1,9	156 10,8	2 0,3	1237	16.6	LC
13	*Muellerianella extrusa* (Scott, 1871)	115 8,3	193 26,3	71 10,6	40 2,3	35 3,9	233 16,1	1 0,2	688	9.2	DD
14	*Macustus grisescens* (Zetterstedt, 1828)	62 4,5	113 15,4	27 4	1 0,1	89 10	88 6,1	8 1,3	388	5.2	LC
15	*Forcipata citrinella* (Zetterstedt, 1828)				206 11,9				206	2.8	NT
16	*Megamelus notula* (Germar, 1830)	15 1,1	12 1,6	1 0,1	33 1,9	31 3,5	98 6,8		190	2.5	NT
17	*Kelisia praecox* Haupt, 1935		7 1	8 1,2		134 15	4 0,3		153	2	VU
18	*Cicadula quadrinotata* (Fabricius, 1794)	7 0,5		4 0,6	48 2,8	19 2,1	22 1,5		101	1.3	LC
19	*Kelisia pallidula* (Boheman, 1847)			24 3,6			58 4		82	1.1	EN
20	*Jassargus sursumflexus* (Then, 1902)	26 1,9	33 4,5	1 0,1	2 0,1		1 0,1		63	0.8	LC
21	*Muellerianella brevipennis* (Boheman, 1847)	5 0,4	2 0,3	2 0,3	15 0,9	12 1,3	21 1,5	1 0,2	58	0.8	LC
22	*Xanthodelphax straminea* (Stål, 1858)	6 0,4		1 0,1		11 1,2	12 0,8		30	0.4	VU
23	*Macrosteles viridigriseus* (Edwards, 1922)				15 0,9				15	0.2	LC
24	*Stenocranus major* (Kirschbaum, 1868)	9 0,6							9	0.1	LC
25	*Cicadula albingensis* Wagner, 1940		7 1						7	0.1	LC
26	*Erzaleus metrius* (Flor, 1861)	5 0,4							5	0.1	LC
27	*Athysanus quadrum* Boheman, 1845						2 0,1		2	<0.1	EN
28	*Streptanus sordidus* (Zetterstedt, 1828)					2 0,2			2	<0.1	LC
29	*Struebingianella lugubrina* (Boheman, 1847)	1 0,1							1	<0.1	VU
**Mesophilic grassland species**											
30	*Jassargus pseudocellaris* (Flor, 1861)				410 23,8	1 0,1			411	5.5	LC
31	*Cicadella viridis* (Linnaeus, 1758)	44 3,2	65 8,8	37 5,5	3 0,2	93 10,4	155 10,7	1 0,2	398	5.3	LC
32	*Neophilaenus lineatus* (Linnaeus, 1758)	45 3,2	70 9,5	90 13,5		91 10,2	26 1,8		322	4.3	LC
33	*Delphacodes venosus* (Germar, 1830)	59 4,2	34 4,6	38 5,7	1 0,1	40 4,5	110 7,6	1 0,2	283	3.8	NT
34	*Arthaldeus pascuellus* (Fallén, 1826)	52 3,7			97 5,6	53 5,9	41 2,8		243	3.3	LC
35	*Dicranotropis divergens* Kirschbaum, 1868				110 6,4				110	1.5	LC
36	*Psammotettix confinis* (Dahlbom, 1850)				79 4,6	3 0,3	1 0,1	1 0,2	84	1.1	LC
37	*Acanthodelphax spinosa* (Fieber, 1866)		1 0,1	2 0,3	58 3,4	12 1,3	6 0,4	1 0,2	80	1.1	LC
38	*Anaceratagallia ribauti* (Ossiannilsson, 1938)				64 3,7				64	0.9	LC
39	*Anoscopus albifrons* (Linnaeus, 1758)		4 0,5		44 2,6	4 0,4	5 0,3		57	0.8	LC
40	*Criomorphus albomarginatus* Curtis, 1833		12 1,6	3 0,4		18 2	11 0,8		44	0.6	LC
41	*Anoscopus flavostriatus* (Donovan, 1799)	2 0,1			9 0,5	13 1,5	15 1		39	0.5	LC
42	*Aphrodes diminuta* Ribaut, 1952		1 0,1	1 0,1	9 0,5	5 0,6	23 1,6		39	0.5	DD
43	*Psammotettix cephalotes* (Herrich-Schäffer, 1834)				27 1,6			1 0,2	28	0.4	NT
44	*Javesella forcipata* (Boheman, 1847)		27 3,7		1 0,1				28	0.4	LC
45	*Javesella dubia* (Kirschbaum, 1868)				26 1,5				26	0.4	LC
46	*Errastunus ocellaris* (Fallén, 1806)	19 1,4			5 0,3				24	0.3	LC
47	*Agallia brachyptera* (Boheman, 1847)		3 0,4	6 0,9	12 0,7				21	0.3	LC
48	*Eupteryx notata* Curtis, 1937				18 1				18	0.2	LC
49	*Athysanus argentarius* Metcalf, 1955	5 0,4	3 0,4	3 0,4		1 0,1	1 0,1		13	0.2	LC
50	*Graphocraerus ventralis* (Fallén, 1806)				13 0,8				13	0.2	LC
51	*Rhopalopyx adumbrata* (C. Sahlberg, 1842)			2 0,3		10 1,1	1 0,1		13	0.2	LC
52	*Anoscopus serratulae* (Fabricius, 1775)				7 0,4				7	0.1	LC
53	*Elymana sulphurella* (Zetterstedt, 1828)				4 0,2				4	0.1	LC
54	*Euscelis incisus* (Kirschbaum, 1858)				4 0,2				4	0.1	LC
55	*Xanthodelphax flaveola* (Flor, 1861)					4 0,4			4	<0.1	EN
56	*Cicadula persimilis* (Edwards, 1920)						3 0,2		3	<0.1	LC
57	*Megophthalmus scanicus* (Fallén, 1806)					3 0,3			3	<0.1	LC
58	*Cercopis vulnerata* Rossi, 1807	1 0,1				1 0,1			2	<0.1	LC
59	*Dicranotropis hamata* (Boheman, 1847)				1 0,1		1 0,1		2	<0.1	LC
60	*Diplocolenus bohemani* (Zetterstedt, 1840)						2 0,1		2	<0.1	LC
61	*Philaenus spumarius* (Linnaeus, 1758)				1 0,1		1 0,1		2	<0.1	LC
**Eurytopic species**											
62	*Deltocephalus pulicaris* (Fallén, 1806)	1 0,1	1 0,1		194 11,3	4 0,4	5 0,3		205	2.7	LC
63	*Macrosteles laevis* (Ribaut, 1927)				39 2,3				39	0.5	LC
64	*Javesella pellucida* (Fabricius, 1794)	1 0,1			23 1,3	1 0,1	2 0,1		27	0.4	LC
65	*Laodelphax striatella* (Fallén, 1826)				3 0,2		1 0,1	1 0,2	5	0.1	LC
**Mesophilic boundary species**											
66	*Macrosteles septemnotatus* (Fallén, 1806)		37 5	4 0,6					41	0.5	LC
67	*Stiroma bicarinata* (Herrich-Schäffer, 1835)				2 0,1	1 0,1	19 1,3		22	0.3	LC
68	*Endria nebulosa* (Ball, 1900)					3 0,3	5 0,3		8	0.1	
69	*Hardya tenuis* (Germar, 1821)	1 0,1				4 0,4	1 0,1		6	0.1	LC
70	*Balclutha calamagrostis* Ossiannilsson, 1961	1 0,1		1 0,1					2	<0.1	LC
71	*Aphrophora alni* (Fallén, 1805)			1 0,1					1	<0.1	LC
72	*Evacanthus interruptus* (Linnaeus, 1758)						1 0,1		1	<0.1	LC
73	*Hyledelphax elegantula* (Boheman, 1847)						1 0,1		1	<0.1	LC
74	*Javesella discolor* (Boheman, 1847)						1 0,1		1	<0.1	LC
**Montane grassland species**											
75	*Verdanus abdominalis* (Fabricius, 1803)				27 1,6	2 0,2	6 0,4		35	0.5	LC
76	*Planaphrodes bifasciata* (Linnaeus, 1758)				12 0,7				12	0.2	LC
77	*Jassargus alpinus* (Then, 1896)							4 0,7	4	0.1	LC
78	*Erythria manderstjernii* (Kirschbaum, 1868)							3 0,5	3	<0.1	LC
79	*Neophilaenus exclamationis* (Thunberg, 1784)						1 0,1		1	<0.1	LC
**Silting zone species**											
80	*Stroggylocephalus agrestis* (Fallén, 1806)			3 0,4		6 0,7	12 0,8		21	0.3	EN
81	*Cosmotettix costalis* (Fallén, 1826)		3 0,4	3 0,4					6	0.1	EN
82	*Limotettix striola* (Fallén, 1806)	1 0,1							1	<0.1	VU
**Xerothermophilic grassland species**											
83	*Eupelix cuspidata* (Fabricius, 1775)		1 0,1	1 0,1		4 0,4			6	0.1	NT
84	*Doratura stylata* (Boheman, 1847)				2 0,1				2	<0.1	LC
85	*Delphacinus mesomelas* (Boheman, 1850)				1 0,1				1	<0.1	VU
86	*Streptanus marginatus* (Kirschbaum, 1858)						1 0,1		1	<0.1	DD
**Hygrophilous forest species**											
87	*Planaphrodes nigrita* (Kirschbaum, 1868)					5 0,6			5	0.1	LC
88	*Macropsis cerea* (Germar, 1837)						2 0,1		2	<0.1	LC
89	*Doliotettix lunulatus* (Zetterstedt, 1840)					1 0,1			1	<0.1	
**Mesophilic forest species**											
90	*Fagocyba cruenta* (Herrich-Schäffer, 1838)							4 0,7	4	0.1	LC
91	*Hesium domino* (Reuter, 1880)				3 0,2				3	<0.1	LC
92	*Ulopa carneae* Wagner, 1955							1 0,2	1	<0.1	EN
**Riparian species**											
93	*Paraliburnia adela* (Flor, 1861)	8 0,6							8	0.1	EN

**Figure 3.**
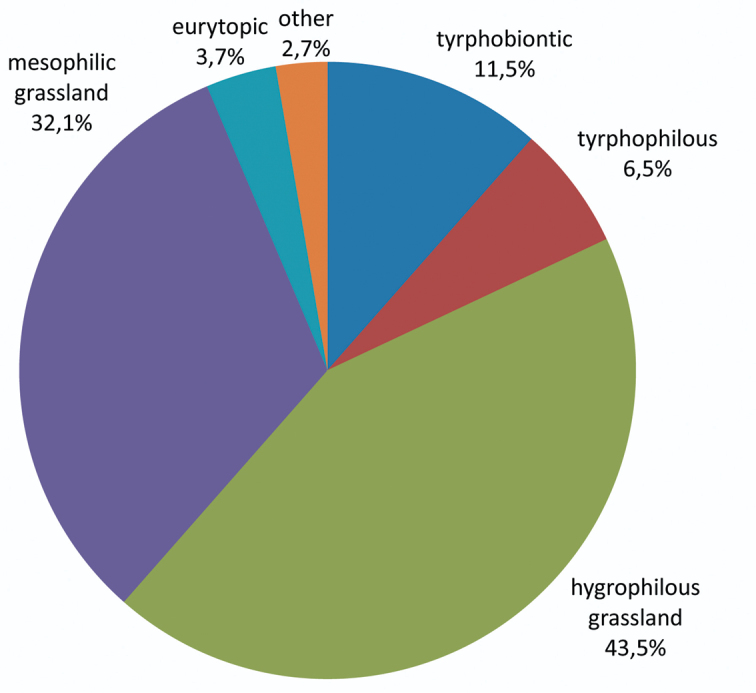
Auchenorrhyncha communities of the Austrian Bohemian Forest peat bogs: Presence of the ecological types (after Holzinger 2009) [percentage of total specimens].

Eleven species are peat bog specialists, i.e. tyrphobiontic or tyrphophilous, according to Nickel et al. (2002). Compared to other wetland areas in Central Europe (see e.g. Schiemenz 1971, 1975, 1976, 1977, Remane and Reimer 1989, Andrew and Rushton 1993, Holzinger and Novotny 1998, Szwedo et al. 1998, Nickel 2002, Nickel and Gärtner 2009, Walter and Nickel 2009, Swierczewski and Blaszczyk 2011), this is a very average number. Peat bog specialists represent 18 % of the total number of individuals collected. The majority of the individuals are hygrophilous and mesophilic grassland species (Figs 3, 5).

**Figure 4.**
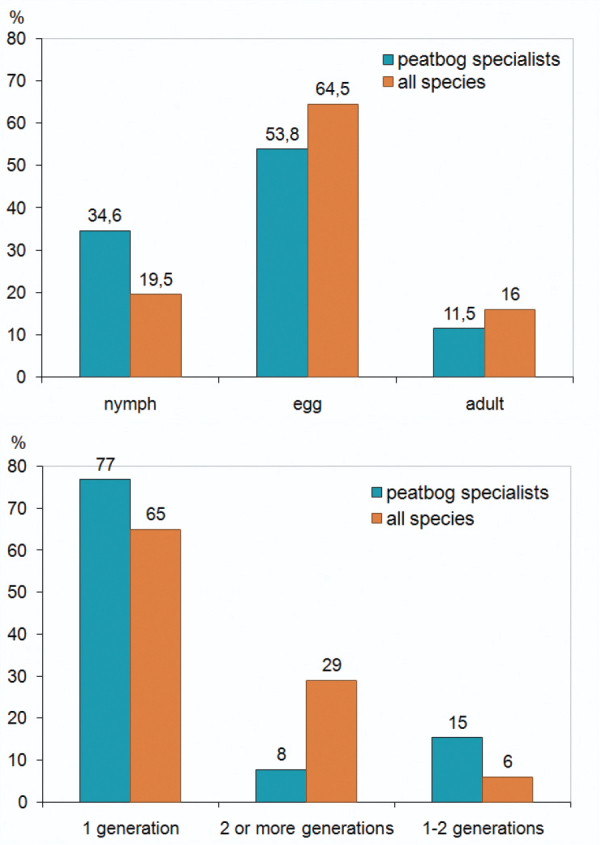
Percentage of the hibernation stages (left) and of generation numbers (right) of Auchenorrhyncha species recorded in Bohemian Forest peat bogs compared to those of the whole Austrian Auchenorrhyncha fauna (data from Holzinger 2009).

Seven species could be found on all sites, among them one peat bog specialist (*Sorhoanus assimilis*). 18 species occur in at least five of the seven sites, among them three more peat bog specialists (*Kelisia vittipennis*, *Paradelphacodes paludosa*, *Oncodelphax pullula*). Almost half of the species (44) were recorded only on one site (among them four peat bog specialists).

### Species composition

The Auchenorrhyncha communities of the peat bogs show higher proportions of univoltine species than the total fauna of Austria. The number of species hibernating in nymphal stages is also higher in peat bogs than in the total fauna of Austria (﻿Fig. 4). This might be caused by comparatively unsuitable conditions (low temperature, high humidity) for Auchenorrhyncha development in these habitats.

The vast majority of the Central European Auchenorrhyncha species is mono- or oligophagous, specialised on one or few host plant species or genera (see Holzinger 2009 for the fauna of Austria). Interestingly, the tyrphophilous and tyrphobiontic Auchenorrhyncha species feeding on *Calamagrostis canescens* and *Molinia caerulea* in Germany (see Nickel 2003) could not be found within this study (whereas the non-tyrphophilous monophagous species are present). These species are missing or very rare in the southern parts of Central Europe and might not (or no longer?) exist in the peat bogs of the Austrian part of the Bohemian Forest (Fig. 6).

**Figure 5.**
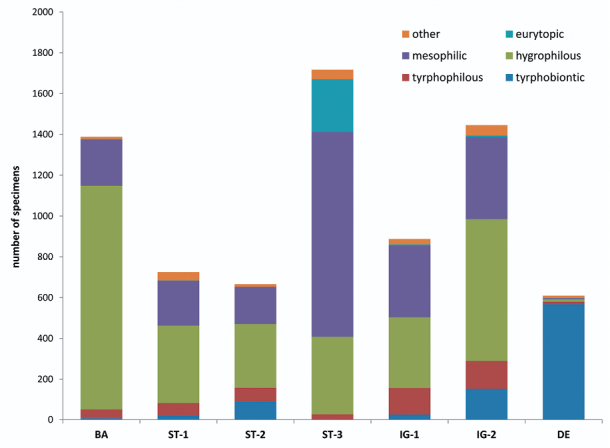
Total number of Auchenorrhyncha collected at the seven peat bog sites. Colours = ecological types (after Holzinger 2009). Abbreviations: BA = Bayrische Au, ST = Stadlau, IG = Moor am Iglbach, DE = Deutsches Haidl. ST-3 is the site with highest human impact (grazing, mowing, dehydratation); DE is a peat bog in much higher elevation than all other sites.

**Figure 6.**
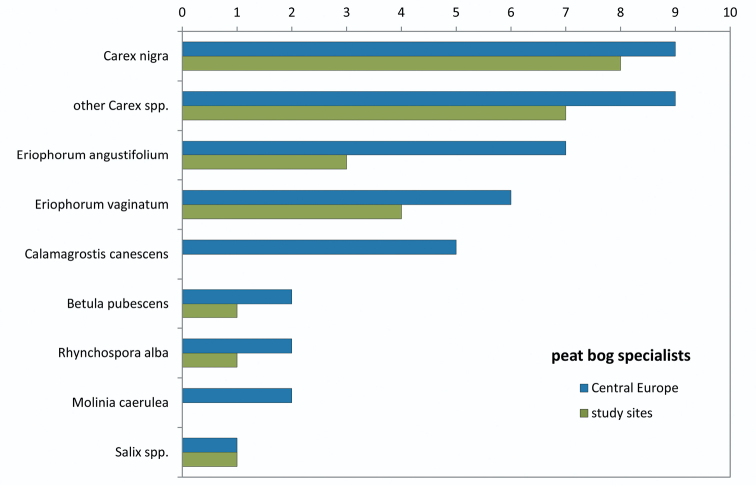
Number of tyrphobiontic and tyrphophilous species specialised on wetland plant species (data from Nickel 2003). Most plant species are utilised by their Auchenorrhyncha hosts also in the study sites, only *Calamagrostis canescens* and *Molinia caerulea* lack their mono-/oligophagous tyrphophilous “species set”.

### Saisonality and densities

**Figure 7.**
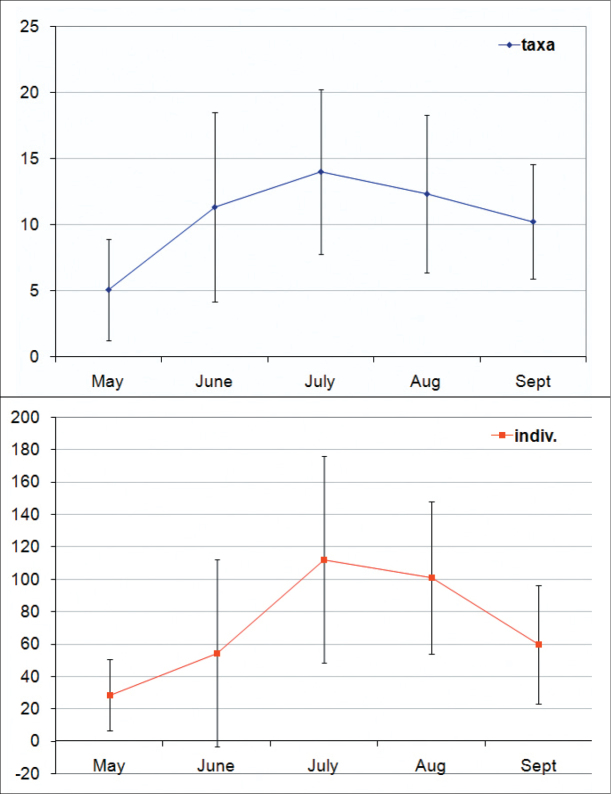
Seasonal mean numbers of Auchenorrhyncha species (left) and adult hopper specimens (=individuals/m²; right) in the peat bogs of the Austrian part of the Bohemian Forest.

The densities of adult Auchenorrhyncha in peat bogs are low in spring (about 10–60 individuals per m²), increase towards July up to 180 (±50) individuals per m² and slowly decreases afterwards (Fig. 7). Disturbed sites have higher species numbers and higher Auchenorrhyncha densities in total but lower numbers and densities in peat bog specialists. The highest proportion of peat bog specialists (almost 95 %) was found in the undisturbed site „Deutsches Haidl“ (Fig. 5).

These Auchenorrhyncha densities of peat bogs are similar to those of other Central European grassland habitats (pastures and meadows: about 50–200 adult specimens/m²; alpine meadows: about 50–100 specimens/m²; ÖKOTEAM unpublished data).

## References

[B1] Andrew CJ Rushton SP (1993) The Auchenorrhyncha of an unimproved moorland in northern England. Ecological Entomology 18: 95-103

[B2] Holzinger WE (1995) Zikaden (Auchenorrhyncha). In: Wieser C Kofler A Mildner P (Eds). Naturführer Sablatnigmoor.Naturwissenschaftlicher Verein für Kärnten: 121-128

[B3] Holzinger WE (2000) Zikaden. In: Naturschutzverein Hörfeld-Moor (Ed) Hörfeld-Moor – Naturjuwel in der Norischen Region, 222–224.

[B4] Holzinger WE (2009) Rote Liste der Zikaden Österreichs (Hemiptera: Auchenorrhyncha). In: Zulka KP (Ed). Rote Listen gefährdeter Tiere Österreichs.Grüne Reihe des Lebensministeriums 14 (3): 41-318

[B5] Holzinger WE Novotny V (1998) Die Zikadenfauna (Homoptera, Auchenorrhyncha) des Pürgschachener Moores (Steiermark, Österreich). Beiträge zur Zikadenkunde 2: 53-56

[B6] Leising S (1977) Über Zikaden des zentralalpinen Hochgebirges (Obergurgl, Tirol), Veröffentlichungen der Universität Innsbruck 107, Alpin-Biologische Studien 9: 1–69.

[B7] Morris MG Clarke RT Rispin WE (2005) The success of a rotational grazing system in conserving the diversity of chalk grassland Auchenorrhyncha. Journal of Insect Conservation 9: 363-374

[B8] Nickel H (2002) Die Zikadenfauna der Hochmoore im Thüringer Wald heute und vor 25 Jahren (Hemiptera, Auchenorrhyncha). Naturschutzreport 19: 116-138

[B9] Nickel H (2003) The leafhoppers and planthoppers of Germany (Hemiptera: Auchenorrhyncha): Patterns and strategies in a highly diverse group of phytophagous insects. Pensoft Publishers, Sofia – Moscow, 460 pp.

[B10] Nickel H Gärtner B (2009) Tyrphobionte und tyrphophile Zikaden (Hemiptera, Auchenorrhyncha) in der Hannoverschen Moorgeest – Biotopspezifische Insekten als Zeigerarten für den Zustand von Hochmooren. Telma 39: 45-74

[B11] Nickel H Hildebrandt J (2003) Auchenorrhyncha communities as indicators of disturbance in grasslands (Insecta, Hemiptera) – a case study from the Elbe floodplains (northern Germany). Abriculture, Ecosystems and Environment 98: 183-199

[B12] Nickel H Holzinger WE Wachmann E (2002) Mitteleuropäische Lebensräume und ihre Zikadenfauna (Hemiptera: Auchenorrhyncha). In: Holzinger WE (Ed). Zikaden – Leafhoppers, Planthoppers and Cicadas (Insecta: Hemiptera: Auchenorrhyncha).Denisia 4: 279–328

[B13] Niedermair M Plattner G Egger G Essl F Kohler B Zika M (2010) Moore im Klimawandel. Österreichische Bundesforste, 24 pp.

[B14] Remane R Reimer H (1989) Im NSG „Rotes Moor“ durch Wanzen (Heteroptera) und Zikaden (Homoptera, Auchenorrhyncha) genutzte und ungenutzte „ökologische Lizenzen“ im Vergleich zu anderen Mooren und der übrigen Rhön. Telma, Beiheft 2: 149-172

[B15] Schiemenz H (1971) Die Zikadenfauna (Homoptera Auchenorrhyncha) der Erzgebirgshochmoore. Zoologisches Jahrbuch zur Systematik, Ökologie und Geographie der Tiere 98: 397-417

[B16] Schiemenz H (1975) Die Zikadenfauna der Hochmoore im Thüringer Wald und im Harz (Homoptera, Auchenorrhyncha). Faunistische Abhandlungen, Staatliches Museum für Tierkunde Dresden 5 (7): 215-233

[B17] Schiemenz H (1976) Die Zikadenfauna von Heide- und Hochmooren des Flachlandes der DDR (Homoptera, Auchenorrhyncha). Faunistische Abhandlungen, Staatliches Museum für Tierkunde Dresden 6 (4): 39-54

[B18] Schiemenz H (1977) Die Zikadenfauna der Waldwiesen, Moore und Verlandungssümpfe im Naturschutzgebiet Serrahn (Homoptera, Auchenorrhyncha). Faunistische Abhandlungen, Staatliches Museum für Tierkunde Dresden 6 (26): 297-304

[B19] Schlosser L (2012) Zoozönotik und Ökologie der Zikadenfauna in Mooren des Böhmerwaldes. Master thesis, Graz, Austria: Karl-Franzens-University, 173 pp.

[B20] Schlosser L Holzinger WE (2012) Bemerkenswerte Zikaden-Nachweise (Insecta, Hemiptera, Auchenorrhyncha) aus Mooren des Böhmerwaldes (Österreich). Linzer biologische Beiträge 44 (1): 845-854

[B21] Steiner GM (1992) Österreichischer Moorschutzkatalog. 4. Auflage. Grüne Reihe des BMUJF 1, 509 pp.

[B22] Stewart AJA (2002) Techniques for sampling Auchenorrhyncha in grasslands. In: Holzinger WE (Ed). Zikaden – Leafhoppers, Planthoppers and Cicadas (Insecta: Hemiptera: Auchenorrhyncha).Denisia 4: 491–512

[B23] Swierczewski D Blaszczyk J (2011) Fauna piewików (Hemiptera: Fulgoromorpha et Cicadomorpha) wilgotnych lasów, lak i torfowisk w poludniowej czesci Wyzyny Wóznicko-Wielunskiej. Ziemia Czestochowska 37: 227-263

[B24] Szwedo J Gebicki C Wegierek P (1998) Leafhopper communities (Homoptera, Auchenorrhyncha) of selected peat-bogs in Poland. Annals of the Upper Silesian Museum (Natural History) 15: 154-176

[B25] Walter S Nickel H (2009) Die Zikadenfauna des Naturparkes Drömling (Sachsen-Anhalt) (Hemiptera, Auchenorrhyncha). Cicadina 10: 71-88

